# Lumbar and para-iliac hernias: an alternative technique

**DOI:** 10.1590/0100-6991e-20213029

**Published:** 2021-06-04

**Authors:** MARIA PESSOLE BIONDO SIMÕES, ALEXANDRE CONTIN MANSUR, SILVANIA KLUG PIMENTEL

**Affiliations:** 1 - Universidade Federal do Paraná, Departamento de Cirurgia - Curitiba - PR - Brasil; 2 - Complexo Hospitalar do Trabalhador, Serviço de Cirurgia Geral - Curitiba - PR - Brasil; 3 - Complexo Hospitalar do Trabalhador, Serviço de Cirurgia Plástica - Curitiba - PR - Brasil

**Keywords:** Hernia, Lumbosacral Region, Herniorrhaphy, Hérnia, Região Lombossacral, Herniorrafia

## Abstract

Lumbar and para-iliac hernias are rare and occur after removal of an iliac bone graft, nephrectomies, retroperitoneal aortic surgery, or after blunt trauma to the abdomen. The incidence of hernia after the removal of these grafts ranges from 0.5 to 10%. These hernias are a problem that surgeons will face, since bone grafts from the iliac crest are being used more routinely. The goal of this article was to report the technique to correct these complex hernias, using the technique of fixing the propylene mesh to the iliac bone and the result of this approach. In the period of 5 years, 165 patients were treated at the complex hernia service, 10 (6%) with hernia in the supra-iliac and lumbar region, managed with the technique of fixing the mesh to the iliac bone with correction of the failure. During the mean follow-up of 33 months (minimum of 2 and maximum of 48 months), there was no recurrence of the hernias.

## RESUMO

Hérnias lombares e para-ilíacas são raras e ocorrem após a retirada de enxerto ósseo ilíaco, nefrectomias, cirurgia aórtica retroperitoneal, ou ainda, após traumas contusos sobre o abdômen. A incidência de hérnia após a retirada destes enxertos varia de 0,5 a 10%. Essas hérnias constituem um problema com o qual os cirurgiões irão se defrontar, uma vez que os enxertos ósseos da crista ilíaca estão sendo usados mais rotineiramente. O objetivo desse artigo foi relatar a técnica para correção dessas hérnias, consideradas complexas, utilizando-se a técnica de fixação de tela de propileno ao osso ilíaco e o resultado dessa abordagem. No período de 5 anos, 165 pacientes foram atendidos no serviço de hérnias complexas, 10 (6%) com hérnia da região suprailíaca e lombar, manejados com a técnica de fixação de tela ao osso ilíaco corrigindo a falha. Durante o seguimento médio de 33 meses (mínimo de 2 e máximo de 48 meses), não houve recidiva das hérnias.

## INTRODUCTION

Lumbar and para-iliac hernias are rare and occur more frequently after the removal of an iliac bone graft, nephrectomies, retroperitoneal aortic surgery, or blunt trauma to the abdomen^1^.

Sakarya et al., in 2003, wrote that they found approximately 300 cases described in the literature^2^. We believe that these hernias occur much more frequently and that they are simply not reported and published. After Sakarya et al., the articles that were published have reported few cases².

The iliac bone is the most common source for obtaining bone grafts in orthopedics, a graft that can be removed from the anterior or posterior iliac crest^3^ ^-^ ^5^. The anterior iliac crest is the preferred site because it is easily accessible, provides abundant spongy bone with a high concentration of osteocompetent cells, and is associated with low morbidity^6^. The incidence of hernia after the removal of these grafts is not well known. Forrest et al. accompanied 82 cases of iliac bone graft removal and found 8 cases of hernias (9.7%)^7^ and Auleda et al. reported 3 cases in 59 graft removals (5%)^8^. Pastore and Sobel reported 0.5% of hernia incidence after the removal of an iliac graft^9^.

Supra-iliac and lumbar hernias are a problem that surgeons face, since bone grafts from the iliac crest are being used more routinely, be it for fracture repair, removal of tumors and even for the increasing incidence of abdominal traumas.

As the number of patients treated by the diverse services is low, it is difficult to find a surgeon with wide experience and even a standard technique for the repair of these hernias.

The purpose of this article is to report on the technique adopted by our incisional hernia service.

## MATERIAL AND METHODS

This study was approved by the Committee for Ethics in Research of our service, under Certificate of Ethical Appreciation and Presentation (CAAE) number 44087121.8.0000.5225.

Patients diagnosed with lumbar and para-iliac hernias comprised the study sample. Demographic data, data on the technique that was employed and the results were gathered. The data collected from medical records were tabulated for descriptive analysis. Due to the rarity of this disease, the number of cases reported did not permit robust statistical analyses.

### Description of the technique and results

At this service, from January 2015 to January 2020, 165 patients underwent surgery. Of these, we found 10 patients with hernia in the supra-iliac and lumbar region, representing 6% of the treated cases. The origin of these hernias was related to the removal of bone graft from the anterior iliac crest in 4 cases, trauma with complex fractures of the iliac bone in 3 cases, nephrectomy in 2 cases and one patient reported blunt lumbar trauma. All of the patients had already been treated at other services and presented recurrences. The number of recurrences was between one and four.

**Figure 1:**
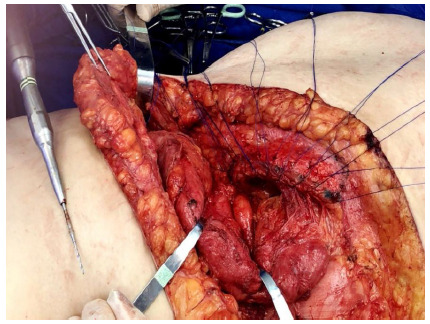
The Bone perforator drill and the polypropylene suture passed through the borehole.

**Figure 2:**
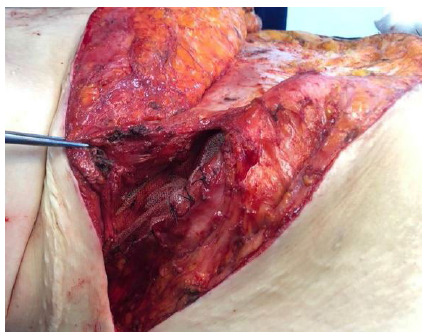
The mesh fixed to the iliac bone.

**Figure 3:**
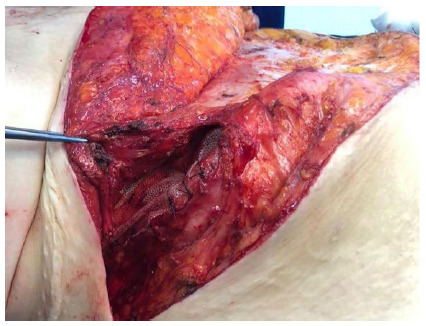
The mesh fixed to the iliac bone under the muscle layer.

The correction was made according to the location of the wall defect. The approach was with open surgery, with an elliptical incision containing the anterior scar, which in this way was resected. The hernial sac and the surrounding structures were identified. For the lumbar hernia a polypropylene mesh of sufficient size was placed that extended beyond the defect by approximately five centimeters in all directions, laterally under the great dorsal and anteriorly under the musculature of the anterolateral wall with separate stitches of zero polypropylene suture. Inferiorly, the tissues next to the iliac were carefully dissected and the mesh was fixed to it. For this purpose, a 17 mm drill was used. During the bone drilling, the drills were kept irrigated so that they would remain cool. Through the borehole, a zero polypropylene suture (Figure 1) passed through the mesh. When the stitches were closed, the mesh was fixed to the iliac (Figure 2). The upper part of the mesh was placed under the anterolateral musculature (Figure 3).

**Figure 4:**
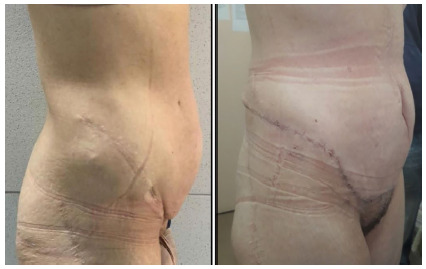
Preoperative and postoperative aspects of the hernia.

The postoperative result showed a correction of the problem, and the follow-up of these patients, with an average of 33 months (minimum of 2 and maximum of 48 months), did not reveal any recurrence. Figure 4 shows the hernia before and after the surgery at the outpatient review.

## DISCUSSION

It is an accepted fact that a successful hernia repair requires an adequate placement of the mesh and appropriate fixing. The fixing of the mesh in the correction of lumbar, supra-iliac and even supra-pubic hernias is recognized as difficult. These defects often have little or no fascia for suture placement and a bony surface comprises one margin of the hernia defect. Furthermore, muscular and fascial retraction does not allow the defect to close^10^.

There is no standard treatment for these hernias. There have been reports of primary repairs, use of tissue flaps, and meshes, including transabdominal and retroperitoneal laparoscopic approaches. Links and Berney published a non-randomized study comparing open and laparoscopic corrections. They showed that the laparoscopic correction required less surgery time and had a lower total cost^11^. Moreno-Egea et al. published another study, also non-randomized, in which they made the same comparison and concluded that the laparoscopic approach was possible^12^. Baird-Gunning et al. highlighted that fact that the minimally invasive option was not always the most adequate option for surgery, especially for major defects and those in which muscular atrophy was present^13^.

Lumbar hernias are limited by the following anatomical points: superiorly by the lower edge of the 12^th^ rib, inferiorly by the iliac crest, medially by the lumbar spinal processes and muscles and laterally by the external oblique muscle. The defects occur in two areas of weakness: the upper lumbar (Grynfeltt) and lower lumbar (Jean Louis Petit) triangles. The upper lumbar triangle is joined superiorly to the 12^th^ rib, the lumbocostal ligament and the inferior posterior serratus muscle; laterally by the internal oblique muscle; and medially by the erector spinae muscle (sacral and ileo-lumbar). The lower lumbar triangle is bordered inferiorly by the iliac crest, laterally by the external oblique muscle and medially by the latissimus dorsi muscle. Lumbar hernias can affect both triangles, but it is in the lower one that, although rare, they are most frequently observed^14^.

Considering the different points where these hernias can manifest, Muysoms et al. classified them as subcostal, flank, iliac and lumbar. Thus, lumbar hernias appear after nephrectomies and surgeries on the aorta and iliac hernias are more closely related to the removal of bone grafts^15^.

The rarity of these hernias means that it is difficult to familiarize oneself with the anatomical limits, contents, nerves and organs involved. Treatment failure are frequent and most likely caused by a lack of experience and the unique features of each of these hernias. According to Carbonell et al., the triangulated nature of the opening of the hernia results in tension on the defect from various angles. The tissues are thin and fibrotic and this makes approximation without tension difficult. Moreover, the edge of the bone is one of the margins of the defect and when the hernia is joined to the iliac or the 12^th^ rib, there is little or no fascia remaining on the bone^1^. Therefore, a recurrence can occur even when a mesh is used due to inadequate fixing of it to the bone structure. It has been seen that there can be diverse primary causes. In cases of bone graft removal, the musculoaponeurotic structures are disconnected from the iliac and reinserted by suture. However, due to the tension in these non-elastic structures, the chance of subsidence is very high, giving rise to a defect that results in an incisional hernia^16^.

The safety and effectiveness of the use of sutures with bone anchoring have been evaluated and published in the last 25 years. Owing to the rare nature of these hernias, it is unlikely that any center will have an adequate number of patients to conduct a controlled and randomized study. Therefore, the experience of several series of cases over time should be considered as evidence for the use of this technique. 

The orthopedic sutures used to reconstruct ligaments have been used to hold prosthetic material on bone surfaces and can be used to repair these hernias. According to Blair et al., the first case published with anchored bone fixing was in 1992^10^. Despite this, few articles can be found, and these have small samples and often report only one case.

Different configurations of bone anchoring are found in the orthopedic arsenal, but in reports found in the medical literature, the most frequently used anchored sutures are the JuggerKnot^®^ and Mitek GII^®^.

Francis et al. described the use of a suture anchored to the iliac crest to reinforce the repair of recurrent laxity following a TRAM flap. The fixation of the mesh to the bone was done with Mitek GII, an anchoring system used in orthopedic surgery^17^.

Patten et al presented two cases in which they used anchoring in the iliac crest with a corkscrew. These patients had already been operated on and presented a recurrence^18^.

Carbonell et al. published 10 cases, of which seven were recurrences. The repair was carried out in ten patients using a polypropylene or polytetrafluoroethylene mesh placed in an extraperitoneal retromuscular position with an overlap of at least 5 cm from the hernia defect. The mesh was fixed with circumferential, transfascial, permanent sutures and inferiorly fixed to the iliac crest by bone anchors with Mitek^1^. Sun et al. reported a patient with several recurrences corrected using the Mitek Anchoring System with a good result^19^.

Blair et al. showed 20 cases of hernias, of which 10 were lumbar, seven suprapubic and three para-iliac. Thirteen were recurrences and the number of recurrences varied from one to seven. They used the Mitek GII System and the follow-up, of up to two years, revealed no recurrences^10^.

Mukherjee and Miller reported the correction of 10 hernias with meshes fixed by anchoring to the iliac crest. The anchored sutures were by DuPuy Inc. (Mitek Sports Medicine, Raynham, MA), with a nickel-titanium alloy. They reported three recurrences in the first 12 months of monitoring (30%)^20^.

Renard et al. reported on perhaps the largest series of cases. They reviewed patients treated at the Robert-Debré University Hospital from January 2009 to January 2015 and found 31 cases. Of these, 19 (61%) were post-nephrectomies, 45% were recurrences and 19% had registered two or more recurrences. They used open retroperitoneal access and placed a mesh inferiorly beyond the psoas muscle, close to the spine and the inferior margin of the rib fixed on the ipsilateral Cooper’s ligaments. Postoperative follow-up was 17.3 to 48.1 months. During this period, there were two recurrences (6.5%)^21^.

Baird-Gunning et al. published the treatment of three patients to whom two meshes were applied: the first in the preperitoneal position fixed to the bone structure with Christmas Tree Bone Anchors, and the second on-lay^13^.

Taking into account that they occur less frequently than other incisional hernias, the high number of recurrences and the technical difficulties involved, the treatment of these hernias is limited to referral hospitals. The collaboration of an orthopedic surgeon who is familiar with bone sutures and the use of anchoring systems is almost always necessary.

It is not always possible for the hernia surgeon and orthopedist to work together at the time of the operation. In many situations, the wall has been manipulated many times and there are problems with the dermo-cutaneous flap. In these cases, the intervention of a plastic surgeon may be necessary. The patients in our service come from the public network and their treatments are expensive. We have sought to have the hernia surgery service and the reconstructive plastic surgery service work jointly.

In the oral and maxillofacial reconstructive surgery, the fixation of bone fragments with interosseous steel wires is used, as well as the placement of plates and screws. Inspired by this, we began to use a variant of anchoring, already used by the authors, making bone paths with a drill and, through them, we inserted non-absorbable polypropylene threads, which have a good degree of elasticity and which, placed through the mesh, allow it to be fixed to the bone margin of the flaw. The use of polypropylene thread rather than steel or even metallic systems such as Christmas Tree Bone Anchors or the Mitek Anchoring System provides better elasticity, avoiding the rupture of the anchoring to the mesh.

Considering the small sample size and the time spent monitoring these patients, it is not possible to discuss recurrences. So far, the results have been very good, with no patients presenting recurrences. Nevertheless, a bigger number of patients with longer follow up is necessary to be more certain. Another important factor is that the technique is easily executed and with polypropylene suture, the cost becomes feasible at any service.
